# Radiosensitization of Hepatocellular Carcinoma through Targeting Radio-Associated MicroRNA

**DOI:** 10.3390/ijms21051859

**Published:** 2020-03-09

**Authors:** Cheng-Heng Wu, Cheng-Yi Chen, Chau-Ting Yeh, Kwang-Huei Lin

**Affiliations:** 1 Department of Biochemistry, College of Medicine, Chang Gung University, Taoyuan 333, Taiwan; rubio9615151@gmail.com; 2 Department of Cell Biology and Anatomy, College of Medicine, National Cheng Kung University, Tainan 70101, Taiwan; csmc8972@hotmail.com; 3 Liver Research Center, Chang Gung Memorial Hospital, Taoyuan 333, Taiwan; chauting@adm.cgmh.org.tw; 4 Research Center for Chinese Herbal Medicine, College of Human Ecology, Chang Gung University of Science and Technology, Taoyuan 333, Taiwan

**Keywords:** radiation, microRNA, DNA damage, apoptosis, cell cycle, liver cancer

## Abstract

Hepatocellular carcinoma (HCC) is the fourth leading cause of cancer-related deaths worldwide. For patients who are resistant to monotherapy, multimodal therapy is a basic oncologic principle that incorporates surgery, radiotherapy (RT), and chemotherapy providing survival benefits for patients with most types of cancer. Although liver has low tolerance for radiation, high-precision RT for local HCC minimizes the likelihood of radiation-induced liver disease (RILD) in noncancerous liver tissue. RT have several therapeutic benefits, including the down-staging of tumors to make them resectable and repression of metastasis. The DNA damage response (DDR) is a cellular response to irradiation (IR), including DNA repair of injured cells and induction of programmed cell death, thereby resulting in maintenance of cell homeostasis. Molecules that block the activity of proteins in DDR pathways have been found to enhance radiotherapeutic effects. These molecules include antibodies, kinase inhibitors, siRNAs and miRNAs. MicroRNAs (miRNAs) are short non-coding regulatory RNAs binding to the 3′-untranslated regions (3′-UTR) of the messenger RNAs (mRNAs) of target genes, regulating their translation and expression of proteins. Thus, miRNAs and their target genes constitute complicated interactive networks, which interact with other molecules during carcinogenesis. Due to their promising roles in carcinogenesis, miRNAs were shown to be the potential factors that mediated radiosensitivity and optimized outcomes of the combination of systemic therapy and radiotherapy.

## 1. Introduction

HCC is the fourth leading cause of cancer-related deaths worldwide. Most patients with HCC have underlying chronic liver disease caused by viral hepatitis and/or nonalcoholic/alcoholic fatty liver disease. These chronic liver diseases are competing comorbidity risks for mortality in many patients [1].

Surgery, such as partial hepatectomy or liver transplantation, remains the gold standard for curative treatment of HCC. Although transplantation is associated with an 84% rate of the two-year overall survival (OS), only 15% to 30% of patients are candidates for transplantation at diagnosis due to tumor extent and underlying liver dysfunction. Other liver-directed therapies (LDTs) evaluated in multidisciplinary settings can include bridge-to-transplant, definitive/curative treatment, and/or palliation, depending on treatment intent. LDTs administered to most patients with non-metastatic HCC over their course of treatment can include radiofrequency ablation (RFA), transarterial chemoembolization (TACE), transarterial radioembolization (TARE), and external beam RT.

In addition to LDTs, the modern strategy of systemic therapy in progressive HCC is a kinase inhibitor Nexavar, which is also known as sorafenib. Sorafenib is a small-molecule multikinase inhibitor against PDGFR, VEGFR and Raf kinases including B-Raf and c-Raf. The Sorafenib HCC Assessment Randomized Protocol (SHARP) Trial randomized controlled trials of sorafenib vs. placebo and have demonstrated modest but significant improvements in OS with sorafenib [2]. In another randomized trial, the Asia-Pacific Trial [3], sorafenib was also associated with an improvement of overall survival. However, the effect of sorafenib-induced overall survival was reduced in the Asia-Pacific Trial as compared with the SHARP trial, due to the increased enrollment of patients with advanced HCC (such as a higher number of intrahepatic tumors or increased extrahepatic disease burden).

For patients who could not tolerate sorafenib or with progression during treatment, there are already early clinical data supporting that multimodal therapy is the next stage of HCC trial studies besides the improvements of the first-line setting. Multimodal therapy is a basic oncologic principle that incorporates surgery, RT, and chemotherapy. These treatments provide survival benefits for patients with most types of cancer. Although guidelines suggest that patients should be treated with single modality therapy during each stage of HCC, the multimodal approach may benefit individual patients, depending on the characteristics of their disease.

As the liver has a low tolerance for radiation, RT was previously not indicated for HCC. However, technological improvements in RT, including three-dimensional conformal RT (3D-CRT), intensity-modulated RT (IMRT), image-guided RT, and stereotactic body RT (SBRT), have enabled the delivery of high-precision RT for local HCC while minimizing the likelihood of RILD in noncancerous liver tissue. These improvements in RT have therapeutic benefits, including the down-staging of tumors to make them resectable and repression of metastasis. Synergic treatment of radiotherapy and target agents is the ongoing field of research. The potential of target drugs as radiosensitizers, especially sorafenib, was supported by retrospective data [4]. In a phase II study, 40 patients who were not candidates for hepatectomy received sorafenib 400 mg twice a day and radiotherapy. 83% of patients completed the course of treatment, a 55% rate of completed or partial response, a 32% rate of the two-year OS, and in-field PFS of 39% in a phase II single-arm study demonstrated that concurrent and sequential sorafenib with radiotherapy was associated with impressive response rates [5]. Recently, Phase I-dose escalation study of combination treatment of sorafenib and SBRT reported the dose-limiting toxicities, aiming to determine dose-limiting toxicities of sorafenib combing with SBRT. The patients with irradiated liver volume (Veff) < 30% were treated by SBRT (39Gy–54Gy) and 400 mg sorafenib daily without any dose-limiting toxicities. In a clinical study, 15 patients were pre-treated with sorafenib for 1 week and followed by RT with sorafenib concurrently. There was a reduction in the liver volume of patients who were treated with sorafenib and RT. This evidence supported the importance of careful assessment before a period of treatment [6].

Although the cooperative interaction of combination therapy enlarged the curative effect, it is also accompanied by increase toxicities or unexpected RILD. The major challenge of combination therapy is the relief or prevention of unexpected side effects and toxicities; this is still the ongoing area of research.

## 2. miRNAs-Mediated Radiosensitivity

### 2.1. miRNAs Involved in the DNA Damage Response

miRNAs are short non-coding regulatory RNAs consisting of about 22 nucleotides. Binding of miRNAs to the 3′-UTR of their target genes controls the expression of these target genes at the post-transcriptional level in a complete/incomplete complementary manner. The gene encoding each miRNA is first transcribed into a pri-miRNA, which is converted to pre-miRNA by the enzyme Drosha. The Exportin-5/Ran–guanosine triphosphate complex promotes the translocation of pre-miRNA from the nucleus to the cytoplasm. Subsequently, the maturation of miRNA is completed by the enzyme Dicer, which catalyzes the removal of the stem-loop structure.

Mature miRNAs target the mRNAs of target genes, regulating their translation and expression of proteins. Individual genes can be regulated by several miRNAs, whereas individual miRNAs can target multiple genes. Thus, miRNAs and their target genes constitute complicated interactive networks, which interact with other molecules during carcinogenesis. These miRNAs can affect various biological processes during tumorigenesis and play an important role in cancer development by influencing tumor cell growth, cycling, differentiation, and apoptosis. Due to their promising roles in carcinogenesis, miRNAs were shown to be the potential factors that mediated radiosensitivity and optimized outcomes of the combination of systemic therapy and radiotherapy.

The DDR is a cellular response to IR, reversing DNA damage and maintaining genome integrity, thereby resulting in cell survival. Although cells have various DDR pathways, their DDRs consisted of three major components: sensors, signal transducers, and effectors.

When DNA damage is induced by IR, poly [ADP-ribose] polymerase-1 (PARP-1) is responsible for DNA single strand breaks (SSBs). The most common form of IR-induced damage, the sensing of DNA double strand breaks (DSBs), is completed by the MRN complex, MRE11/RAD50/NBS1. After they sense damage, ataxia-telangiectasia mutated (ATM) and ATM and Rad3-related (ATR), the pivotal kinases of the DDR that trigger downstream DDR cascades, can be used to detect DNA damage. Generally, ATR regulates SSBs, whereas ATM activates the DDR involving DSBs.

As DSB is a major type of IR-induced damage, we focused on the miRNAs that regulate ATM. miR-203a-3p targeting ATM was found to repress the proliferation, migration, and invasion of ovarian cancer cells and to facilitate their apoptosis through the Akt/GSK-3β/Snail signaling pathway [7]. Moreover, up-regulation of miR-18a-5p inhibited the levels of expression of ATM and pATM (phospho S1981) and enhanced the radiosensitivity of A549 and CD133^+^ stem-like cells [8]. Enhanced miR-203 also inhibited ATM activity and suppressed the ATM-Snail pathway, increasing E-cadherin, and thereby preventing the migration and invasion of gastric cancer cells [9].

ATM was found to be the direct target of miR-203 in colorectal cancer (CRC) cells. Mutation of the putative miR-203 binding site in the 3′-UTR of ATM mRNA abrogated the inhibitory effect of miR-203 on ATM, with miR-203 inducing oxaliplatin resistance in CRC cells [10]. Overexpression of miR-18a was found to down-regulate ATM expression by directly targeting the ATM-3′-UTR, reducing DNA damage repair activity and increasing cellular radiosensitivity to IR treatment [11]. miR-421 was found to suppress ATM expression in LA-N-1 and LA-N-5 neuroblastoma cells in vitro by targeting the 3’-UTR of ATM transcripts, with ATM 3’-UTR rescuing the radioresistance phenotype caused by miR-421 [12].

ATM is responsible for the phosphorylation of histone H2AX, which mediates DSBs’ repair and recruits DNA repair proteins to the site of damage. Knockdown of miR-328 was reported to up-regulate histone H2AX expression and increase the number of TUNEL-positive cells in vitro. Down-regulation of miR-328 decreased the incidence of lung cancer induced by *Chlamydia pneumoniae*, reduced tumor volume, increased caspase-3 activity, and facilitated tumor cell apoptosis in vivo. These findings revealed that histone H2AX is the target of miR-328 and participates in lung cancer regulation, and that reductions in miR-328 levels promote apoptosis of lung cancer tissue [13]. miR-138 was shown to directly target the 3′-UTR of histone H2AX, with overexpression of miR-138 inhibiting homologous recombination and enhancing cellular sensitivity to multiple DNA damaging agents. Expression of histone H2AX in miR-138 overexpressing cells attenuated miR-138-mediated sensitization to radiotherapy and to chemotherapy with DNA damaging agents [14]. 

Activation of ATM and histone H2AX also involves RAD51, DNA-dependent protein kinase (DNA-PK), breast cancer susceptibility gene 1 (BRCA1) and breast cancer susceptibility gene 2 (BRCA2), all of which play essential roles in completion of DNA damage repair. Cells possess two major DNA repair pathways: homologous recombination repair (HRR) and non-homologous end-joining (NHEJ). DNA-PK is a pivotal factor for NHEJ in the DDR. miR-101 was reported to reduce the expression of ATM or DNA-dependent protein kinase, catalytic subunit (DNA-PKcs), the catalytic subunit of DNA-PK by binding to their own 3′-UTR sequences, sensitizing the GBM cell line U87MGD to radiation [15]. miR-101 had similar effects in pancreatic cancer cells [16]. RAD51 was found to participate in HRR to avoid illegitimate recombination events leading to genetic instability. miR-34a was shown to regulate RAD51 by directly binding to its 3’-UTR, controlling HR and promoting radiosensitivity in NSCLC cells [17]. In addition, miR-155 was reported to regulate DNA repair activity and sensitivity to IR by repressing RAD51 in breast cancer cells [18], and miR-34a/b/c-5p was shown to directly target the RAD51 mRNA 3′-UTR or indirectly inhibit RAD51 expression via the p53 signaling pathway, indicating that miR-34s overexpression reduces the efficiency of HR repair and induces DSBs by down-regulating RAD51 expression [19].

BRCA1 was shown to be involved in several important cellular pathways, including DNA damage repair, chromatin remodeling, and checkpoint activation, thereby affecting the outcomes of cancer therapy. Overexpression of miR-212 down-regulated BRCA1 expression, and knockdown of BRCA1 attenuated IR-induced apoptosis [20]. Increased expression of miR-7-5p was found to further repress PARP-1 and BRCA1 expression in lymphoblastoid cells, increasing apoptosis and inhibiting cell proliferation [21]. miR-9 was also reported to directly regulate BRCA1 expression in ovarian cancer, having more therapeutic potential than treatment with PARP-1 inhibitors [22]. 

BRCA2 is a major component of the homologous recombination DNA repair pathway. Testing of interactions between BRCA2 and miR-19a/miR-19b in 15 cell lines derived from pancreatic, breast, colon, and kidney cancers showed that overexpression of these two miRNAs reduced BRCA2 expression by directly binding to its 3′-UTR [23]. In addition, miR-1245 was found to directly bind to the 3’-UTR of BRCA2 and suppress its translation, impairing HR-mediated repair, reducing the number of DNA damage-induced RAD51 nuclear foci, and rendering cells hypersensitive to γ-irradiation (IR). Furthermore, the c-myc protein was observed to up-regulate miR-1245 expression via direct binding to the miR-1245 promoter, down-regulating BRCA2 and reducing the efficiency of HR [24].

### 2.2. miRNAs Involving the Cell Cycle

The cell cycle in humans consists of four phases, in which transitions were regulated by cell cycle checkpoints through cyclins and cyclin-dependent kinases (CDKs). Deregulation of the cell cycle was frequently observed in carcinomas and affected by treatments such as radiotherapy. IR-induced DNA damage can trigger cell cycle checkpoints, arresting the cell cycle and allowing time for DNA damage repair. Thus, inhibiting the transition of other working checkpoints prevents cell cycle progression, allowing more tumor cells to be killed by radiotherapy.

Repair of DNA damage allows cells to re-enter the cell cycle. If damage is not repaired, however, these cells will progress to apoptosis or senescence, preventing tumorigenesis resulting from genomic instability. Chk1 and Chk2 are the major cell cycle checkpoint inhibitors. Chk1 is a direct target of miR-195, with down-regulation of miR-195 sensitizing HCT-116 cells to treatment with 5-FU [25]. In addition, miR-200c was reported to repress Chk1 through targeting of LINC02582 [26].

Cdc25a contains highly conserved domains for dual-specificity phosphatases, which dephosphorylate and activate cyclin-dependent kinase complexes. Irradiation and other DNA-damaging agents activate the signal cascade from ATM and ATR to Chk1 and Chk2, whereas activation of Cdc25a by Chk-1 initiates the procedure leading to cell-cycle arrest. This Cdc25a axis is thought to be the main pathway of cell cycle arrest independent of p53 and a critical regulator of the cell-cycle checkpoint in response to DNA damage caused by IR, oxidative stress, and other DNA-damaging agents. miR-21 knockdown was found to enhance RT-induced GBM cell growth arrest and apoptosis by directly or indirectly modulating Cdc25a levels, thereby reducing G2/M cell cycle accumulation [27].

Irradiation of LNCaP cells has been shown to induce miR-449a, which targets Cdc25a both directly and indirectly. Suppression of Cdc25a reduced Rb phosphorylation, E2F1 expression and the Cdc2/cyclin B1 complex, leading to G2/M phase arrest and sensitizing cancer cells to ionizing radiation [28].

### 2.3. miRNAs Involved in Apoptosis

p53 is a multifunctional tumor suppressor capable of activating transient cell cycle checkpoints and accelerating DNA repair (e.g., rejoining of DNA double strand breaks; DSBs) to promote survival, and inducing growth arrest through stress-induced premature senescence (SIPS) or activating programmed cell death (e.g., apoptosis) to eliminate highly injured cells from the proliferating population [29]. SIPS is a sustained growth-arrested state resembling replicative senescence, triggered by DNA-damaging agents. It is a prominent reaction of cells that express wild-type p53 to genotoxic stress (ionizing radiation) [29,30,31]. Failure of cells to properly engage p53-mediated responses (checkpoints, SIPS, apoptosis) under stressful conditions can result in the creation of polyploid/multinucleated giant cells (MNGCs). Such giant cells exhibit resistance to genotoxic agents and can give rise to tumor repopulating progeny that has been reported for several carcinomas [32,33,34,35]. For targeting these dormant cancer cells, a recent literature reported that down-regulating the Bcl-XL/Bcl-2 pathway in multinucleated colon carcinoma cells (HCT116) results in the rapid death of multinucleated giant cells through the treatment of ABT-263 (a small-molecule inhibitor of Bcl-X, Bcl-2 and Bcl-w) [36].

p53 can activate apoptotic signaling through its proline-rich region or inducing the expression of pro-apoptotic proteins such as PUMA (interaction with Bcl-2 and Bcl-Xl), Bax and NOXA (resulting cytochrome c releasing from mitochondria) [37]. Concurrently, p53 transcriptionally activates DNAJB9 and inhibits apoptosis through direct interaction with it. p21 can also be activated by p53, preventing p14^ARF^-modulated MDM2 degradation and induction of WIP1 (a phosphatase targeting p53 and its upstream kinase) [38].

The final outcome of p53 signaling in response to genotoxic stress in terms of sustained growth arrest or apoptotic cell death depends on the genetic background of the cells’ several factors and genotoxic stress. In most human cells, exposure to moderate doses of ionizing radiation, UV, or chemotherapeutic drugs promotes a high degree of SIPS rather than apoptosis [39]. Stress-induced growth arrest in cancer cells results in the emergence of cancer repopulating progeny. Selective targeting of those growth-arrested cancer cells from arrest to apoptosis through lowering the apoptotic threshold was a potential strategy for the effectiveness of genotoxic cancer therapy [40].

Two basic apoptotic signaling pathways have been described to date, the intrinsic and extrinsic apoptotic pathways. The intrinsic apoptotic pathway is triggered by numerous stimuli, including DNA damage and oxidative stress, leading to the formation of apoptosomes, which are composed of apoptotic protease activating factor 1 (Apaf-1), cytochrome c and procaspase-9. p53 modulates the expression of several members of the Bcl-2 family, including anti-apoptotic Bcl-2, Bcl-xL, Mcl-1, and pro-apoptotic Bax, Bak, Bim, Bid. Bcl-2 family mediates apoptotic response through the interplay of each other, thus facilitating mitochondrial membrane permeabilization and the release of cytochrome c. miR-365 was found to directly target pro-apoptotic protein Bax and adaptor protein Src Homology 2 Domain Containing 1 (SHC1), inducing resistance of gemcitabine in pancreatic cancer cells [41]. Bak1 was targeted by miR-125b, leading to suppression of Taxol-induced apoptosis and increased resistance to Taxol [42]. miR-133a was found to be down-regulated in osteosarcomas, with its expression correlating with tumor progression and patient prognosis. Expression of miR-133a could reduce cell proliferation, promote cell apoptosis, and suppress tumorigenicity by targeting Bcl-xL and Mcl-1 and reducing their expression [42]. Bcl-xL was also directly targeted by miR-491, contributing to miR-491-induced apoptosis. Treatment with miR-491 also suppressed tumor growth in vivo, suggesting that miRNAs may have therapeutic potential through regulation of Bcl-xL in CRC cells [43]. Bcl-xL was found to be a direct target of miR-608, with expression of miR-608 inducing apoptosis in chordoma cells [44]. miR-148a can induce apoptosis in CRC cells through posttranscriptional repression of Bcl-2 [45]. Moreover, miR-24-2 was shown to target the antiapoptotic gene Bcl-2, with overexpression of miR-24-2 inducing apoptosis by down-regulating Bcl-2 expression [46]. Down-regulation of miR-204 was found to correlate with increased expression of Bcl-2 protein in gastric cancers (GCs). Moreover, miR-204 increased the sensitivity of GC cells to treatment with 5-fluorouracil and oxaliplatin by targeting Bcl-2, whereas restoration of Bcl-2 protein counteracted this response to 5-fluorouracil [47]. Expression levels of miR-15a and miR-16-1 correlated negatively to Bcl-2 expression in chronic lymphocytic leukemia (CLL), which was characterized by malignant B cells with overexpression of Bcl-2. Both microRNAs repressed Bcl-2 at a posttranscriptional level. Bcl-2 down-regulation by these microRNAs was found to induce apoptosis and could be used for therapy of Bcl-2-overexpressing tumors [48].

Apoptotic protease activating factor-1 (Apaf-1) was repressed by miR-23a/b and miR-27a/b, which regulate neuronal apoptosis [49], and by miR-24a in the developing neural retina [50]. 

The extrinsic pathway of apoptosis is initiated by the binding of death ligands (Fas ligand (FasL), TNF-related apoptosis-inducing ligand (TRAIL), TNF-α, and TNF-like weak inducer of apoptosis (TWEAK) etc.) to death receptors belonging to the TNF receptor (TNFR) superfamily. These death ligand/death receptor complexes activate pro-caspase-8, with the resultant caspase-8 triggering the activation of pro-caspase-3, inducing apoptosis. FasL expression induced cancer cell apoptosis after gemcitabine treatment, an apoptotic process partially counteracted by the ectopic expression of miR-21 [51]. miR-20a suppressed Fas expression in osteosarcoma, contributing to the ability of osteosarcoma cells to metastasize to the lungs [52].

miR-146a, which targets Fas, was shown to be involved in the pathogenesis of autoimmune lymphoproliferative syndrome (ALPS) [53], whereas the miR-196b-induced reduction in FAS expression significantly promoted leukemogenesis [54]. miR-25 was shown to target TRAIL/death receptor-4 (DR4) complexes, suppressing TRAIL-induced apoptosis, whereas antagonism of miR-25 was found to sensitize cells to apoptotic death [55]. TNF-α overexpression was found to induce apoptosis, whereas ectopic expression of miR-181c partially protected neurons from cell death [56]. Ectopic expression of miR-187 [57], miR-34a-5p and miR-34a-3p [58] consistently and selectively reduced TNFα-induced apoptosis in monocytes. Fas-associated death domain-containing protein (FADD) and caspase-3 were found to be the key factors in FasL–Fas signaling. Ectopic expression of miR-155 in human NP cells repressed FADD and caspase-3 expression; whereas knockdown of miR-155 restored their expression. Ectopic expression of miR-128a conferred Fas-resistance on Jurkat cells by directly targeting FADD. Thus, antagonizing miR-155 [59] and miR-128a [60] can target Fas-mediated apoptosis. Although caspase-3 was well-characterized as an apoptotic factor in both intrinsic and extrinsic pathways, many literatures also report that caspase-3-mediated secretion of PGE2 and tumor repopulation is associated with these stress-induced cells [61]. However, this repopulation could be abrogated by treatment with a PGE2-neutralizing antibody or PGE-2 inhibitor (such as celecoxib, a pharmacological inhibitor which repressed the production of PGE-2 through targeting cyclooxygenase-2) [62].

### 2.4. miRNAs Involved in Radio-Related Signal Transduction Pathways

Four well-recognized pathways have been confirmed as playing roles in radiotherapy and to be closely associated with radiosensitivity. Three of these pathways, the PI3K/Akt pathway, the mitogen-activated protein kinase (MAPK) pathway, and the pathway involving nuclear factor-kappa B (NF-κB) and transforming growth factor-β (TGFβ), are regarded as being associated with radiation. Radiation-induced activation of the PI3K/Akt and MAPK/ERKs pathways was shown to suppress intrinsic apoptosis through repression of downstream target genes, including *Bad* and *Bim*. In contrast, the NF-κB/ TGFβ pathway enhances intrinsic apoptosis by increasing the expression of the antiapoptotic protein, Mcl-1. By affecting apoptosis, these pathways contribute to tumor radioresistance.

In addition to participating in cell death, the PI3K/Akt and MAPK/ERK pathways are activated by radiation and affect DNA damage repair pathways by influencing DNA-PKcs, the major components of the NHEJ pathway, thereby modulating tumor radioresistance. Moreover, epidermal growth factor receptor and insulin-like growth factor receptor were found to be directly involved in the process of NHEJ after their translocation to the nucleus. These growth factors also affect DNA-PKcs activities, contributing to tumor radioresistance. In addition to these three pathways in DDR, the TGFβ pathway contributes to two major repair pathways in DDR, the NHEJ and HR pathways, by activating the *ATM* gene.

The importance of radio-related signal transduction pathways has led to therapeutic approaches that enhance tumor radiosensitivity and reduce tumor radioresistance. Molecules that block the activity of proteins in signal transduction pathways have been found to enhance apoptosis and reduce DNA damage repair, increasing tumor radiosensitivity and radiotherapeutic effects. These molecules, including antibodies, kinase inhibitors, siRNAs and miRNAs, have been used to suppress the function of crucial signaling pathways, such as those involving PI3K, Akt, MAPK, NF-κB and TGFβ. PTEN is a tumor suppressor gene that acts upstream of Akt. The miRNAs miR-21, miR-26, miR-486, miR-221/222, and miR-216a/217 control the activation of Akt by regulating the expression of PTEN. In addition, miR-155, miR-205 and miR-375 control Akt activation by regulating the expression of the SH2-containing inositol 5′-polyphosphatase (SHIP) and PDK1 genes. Moreover, miR-126 and miR-320 control PI3K expression, affecting the downstream activities of PIP3, and altering the levels of expression of total and phosphorylated Akt protein [63]. miR-486 can directly target PTEN and Foxo1a, contributing to Akt phosphorylation, with phosphorylated Akt shown to phosphorylate GSK3β, a negative regulator of Foxo1a, ensuring constant activation of the PI3K/Akt pathway [64].

The miRNAs, miR-221 and miR-222, have been found to regulate the viability, apoptosis, cell cycle progression and invasive ability of gastric cancer cells by down-regulating PTEN expression and enhancing Akt phosphorylation. Suppression of miR-221 and miR-222 may represent a novel therapeutic strategy for gastric cancer through the PI3K/Akt pathway [65]. The miR-17-92 cluster, composed of miR-17-5p, miR-17-3p, miR-18a, miR-19a, miR-20a, miR-19b, and miR-92-1, has been linked to cancer pathogenesis. miR-17-5p was shown to be overexpressed in HCC, resulting in the suppression of the p38 MAPK pathway through the miR-17-5p/E2F1/Wip1 axis [66].

Transcriptional active heterodimer of NF-κB is repressed by IκB, and IκB degradation is regulated by IκB kinase complex (IKK) through phosphorylation. NF-κB signaling is mediated by various miRNAs through targeting IκB or IKK. IκBα is repressed by miR-668 in breast cancer; IκBβ is suppressed by miR20a in gastric cancer; IKKα is negatively modulated by miR-156-5p in colorectal cancer; IKKβ is down-regulated by miR-218 in glioma cancer, miR-199a in ovarian cancer, miR-451 in HCC and miR-429 in cervical cancer. miR-223 targeted both IKKα and IKKβ in lung cancer. Moreover, NF-κB repressing factor NKRF is targeted by miR-301a, repressing the activity of the p50 subunit of NF-κB [67].

### 2.5. Radiation-Associated miRNAs in HCC

HCC is usually diagnosed at a late stage; most of the patients will not be the candidates for hepatectomy and liver transplantation. In addition, the impairment of liver function also restricts the efficacy of systemic therapy such as chemotherapy or targeted therapy due to intolerable side effects. Sorafenib exerts its anti-tumor functions mainly through repressing tumor cell proliferation and angiogenesis. However, HCC patients could acquire resistance, happening within 6 months. The high incidence of sorafenib resistance has become a limiting factor in its clinical application; combining with radiotherapy is an ideal option for current treatment. However, the combination treatment of sorafenib and radiotherapy increases toxicity; minimizing toxicity through sensitizing tumors to radiotherapy is an ongoing area of research.

Replication protein A3 (RPA3) can affect DNA recombination, repair, and replication, as well as cell cycle checkpoints. Up-regulation of RPA3 expression has been reported to promote HCC progression. miR-146a-5p can suppress the proliferation of HCC cells and their radiosensitivity and apoptosis by activating DNA repair pathways and inhibiting RPA3 [68]. 

WEE1 is a tyrosine kinase that acts to regulate the cell cycle. Down-regulation of WEE1 was found to enhance the radiosensitivity of HCC cells, as evidenced by reduced survival and enhanced apoptosis of Huh7 and PLC5 cells. WEE1 was shown to be a direct target of miR-101-3p. NEAT1_2 was shown to up-regulate WEE1 through sponging by lncRNA miR-101-3p and down-regulation of lncRNA NEAT1_2, resulting in the radiosensitization of HCC cells [69]. PTEN deficiency was thought to be the leading cause of hyperactivation of the PI3K/Akt signaling pathway. Forced expression of miR-20a activated the PI3K/Akt signaling pathway, an effect reversed by the PI3K tyrosine kinase inhibitor LY294002, indicating the importance of the miR-20a mediated PI3K/Akt pathway in radioresistance [70].

It has been reported B-cell-specific Moloney leukemia virus insertion site 1 (Bmi-1) was correlated with poor overall survival in various cancer and involved in tumor initiation, progression and radiosensitivity through mediated transcription of p16^Ink4a^ and p14^ARF^ [71,72]. The bioinformatics prediction and the result of luciferase assay identified that Bmi-1 was directly targeted by miR-203, which radiosensitized cancer cells through repression of Bmi-1, ectopic expression of Bmi-1 without 3′-UTR could reverse the improvement of radiosensitivity by miR-203 [73].

F-box and WD repeat domain containing 7 (FBXW7), a member of the F-box protein family, which has been reported to function as a tumor suppressor in various human cancers. Notably, the former findings indicate that FBXW7 is implicated in prognosis and induction of apoptosis through repression of YAP expression in HCC [74]. FBXW7 was a direct target of miR-106a and its interference reversed the regulatory effect of miR-106a abrogation on migration, invasion, and radiosensitivity in HCC cells [75].

The SET domain bifurcated 1 (SETDB1) is a methyltransferase that mediates the transcriptional inhibition of euchromatin through catalyzing the methylation of histones, mainly H3K9me3. SETDB1 is one of the highly expressed genes of various malignant tumors in the TCGA database and related to the progression of carcinoma. In previous literature, SETDB1 forms a complex with p53 and catalyzes the dimethylation of p53K370me2, leading to degradation of p53 via MDM2 in HCC cells [76]. *SETDB1* was a direct target gene of miR-621, enhancing the radiosensitivity of HCC cells and activating the p53-signaling pathway via inhibiting the expression of SETDB1 as a radiosensitizer in HCC [77].

It has been reported that the down-regulation of miR-26b in HCC is correlated with cancer progression and poor prognosis. Erythropoietin-producing hepatocellular carcinoma A2 (EphA2) mediates tissue renewal and embryonic morphogenesis, but its dysregulation has been associated with tumorigenesis and metastasis in many cancers [78]. The expression profiles and the result of dual reporter assay revealed the interrelationship of miR-26b and EphA2. Transfection of miR-26b mimics or EphA2-shRNA leading to radiosensitization of 97H HCC cells and overexpression of EphA2 can protect 97H HCC cells from irradiation [79].

As aforementioned, activation of anti-apoptotic proteins and/or suppression of pro-apoptotic proteins could lead to apoptosis resistance in cancer cells. Several pieces of literature have been reported that miRNAs targeting the anti-apoptotic genes (such as Mcl-1, Bcl-xL) can enhance the sorafenib-induced apoptosis. miR-193b can sensitize the HBV-positive HCC cells to sorafenib through targeting Mcl-1 directly [80]. Let-7 miRNA is negatively correlated with Bcl-xL in HCC tissues and sensitizes hepatoma cells to sorafenib-induce apoptosis through targeting 3′-UTR of Bcl-xL [81]. 

Besides, there are miRNAs promoting sorafenib resistance of HCC through the repression of pro-apoptotic genes. The aberrant expression of miR-221 has been reported in HCC; overexpression of miR-221 in chemical-induced HCC rat model and xenograft mouse model were leading to sorafenib resistance through caspase-3 modulation [82]. Moreover, liver-specific miR-122 is repressed in HBV-related HCC, UDP-N-acetyl-α-d-galactosamine: polypeptide N-acetylgalactosaminyltransferase-10 (GALNT10) is the target by miR-122. Repression of GALNT10 decreases the expression of Mcl-1 and Bcl-2, increasing sorafenib and doxorubicin resistance of hepatoma cells [83].

## 3. Discussion

**Figure 1:**
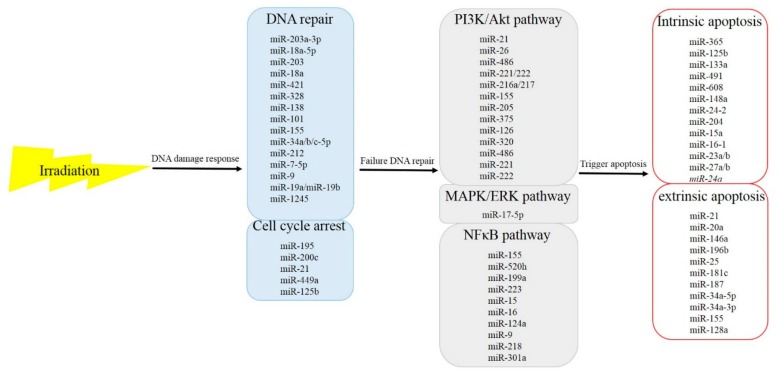
microRNAs that respond to irradiation and the functional pathways in which they participate.

As the incidence of HCC increases, the need for study and effective treatment options have become even more pivotal. Sorafenib remains the recommended first-line effective systemic targeted agent for patients with metastatic or locally advanced HCC who are not candidates for local therapies, but outcomes remain poor. In addition to systemic therapy, aggressive local therapy, like radiotherapy, has emerged as a safe and effective treatment option in patients with advanced disease; a randomized trial of sequential radiotherapy with sorafenib is ongoing. The potential for synergy of radiotherapy and target agents remains an area of research. As aforementioned, miRNAs play roles in the radiosensitivity of cancer cells (Figure 1). These miRNAs affect cell survival through the signal cascades of DNA damage response from upstream to downstream. These findings suggest that miRNAs may be an ideal clinical target to modulate cellular radiosensitivity, and that the regulatory roles of miRNAs in tumor radiosensitivity may be useful clinically in the near future. The potential role of miRNAs as radiosensitizers in the combination of radiotherapy and sorafenib raises the possibility of minimizing RILD in HCC patients and improves treatment efficacy, improving the killing rate of radiation and enhancing the OS of advanced HCC patients. Although these miRNAs have shown activity in vitro, future studies are needed to evaluate the effects of miRNAs on radiosensitivity in vivo. Improvements in miRNA delivery techniques have raised the possibility of the successful development of a new class of radiosensitizers for cancer patients.
